# The Effect of Comb Cell Size on the Development of *Apis mellifera* Drones

**DOI:** 10.3390/life14020222

**Published:** 2024-02-04

**Authors:** Lifu Zhang, Linxin Shao, Muhammad Fahad Raza, Richou Han, Wenfeng Li

**Affiliations:** 1 Guangdong Key Laboratory of Animal Conservation and Resource Utilization, Guangdong Public Laboratory of Wild Animal Conservation and Utilization, Institute of Zoology, Guangdong Academy of Sciences, Guangzhou 510260, China; zhanglf@giz.gd.cn (L.Z.); shaolx@giz.gd.cn (L.S.); fbiuaf@giz.gd.cn (M.F.R.); hanrc@giabr.gd.cn (R.H.); 2 Department of Entomology, College of Plant Protection, South China Agricultural University, Guangzhou 510640, China; 3 College of Animal Sciences (College of Bee Science), Fujian Agriculture and Forestry University, Fuzhou 350002, China

**Keywords:** honeybees, development, cell size, drones, *Apis mellifera*

## Abstract

The growth and development of honeybees are influenced by many factors, one of which is the cell size of the brood comb. Larger worker bees can be obtained by being raised in bigger cells. However, whether cell size has the same effect on drone development is still unknown. Here, using 3D-printed foundations, we observed the development of drones kept in comb cells of different sizes from the late larval stage through eclosion. The results showed that drones in larger cell-size combs had heavier body weights, longer body lengths, and larger head widths, thorax widths, and abdomen widths compared to those in smaller cell-size combs. Furthermore, regardless of developmental stages, the drones’ body weights increased linearly with the comb’s cell size. However, the other morphological changes of drones in different developmental stages were out of proportion to the cell-size changes, resulting in smaller cells with a higher fill factor (thorax width/cell width). Our findings confirm that comb cell size affects the development of honeybees; drones become bigger when raised in large cells.

## 1. Introduction

As a eusocial insect, honeybees live like superorganisms that form social groups with cooperation, ensuring that not a single one can survive independently [1]. This association includes cooperative brood care, reproductive division of labor, and parasite defense [2]. The task of drones and queens is mating, while workers have other diverse jobs, such as creating nests, breeding, and collecting pollen [3]. Honeybees play an essential role in the ecosystem. More than 90% of plants in the world need to pollinate through animals, and bees are the primary pollinators. In addition, bees are essential to agriculture production, about 75% of crops benefit from animal pollination [4].

People used to think that larger honeybees may be more efficient for pollination and honey collection, and how to raise larger honeybees has become an interesting topic. Baudoux was the first person to consider using an artificial foundation to change the size of honeybees, and he demonstrated that the size of the bee is in proportion to the size of the cell [5]. Many scholars have researched brood cell size and the changes in honeybees. Pensieri confirmed that the new generation had increased 10% in body size and weight when he used the comb with a size of 5.42 mm, which was larger than that built naturally [6]. Grout studied the relationship between cell size and the morphometry of honeybees. He found that the size of adult bees that emerged from enlarged brood cells was significantly larger than that from the comb with a standard foundation. He concluded that enlarged brood cells affect the size of honeybees, and the increases in the bee’s body size are almost proportional to the percentage increases in brood cell size [7]. McMullan et al. undertook the study to figure out whether current bees could return to the cell size of the 1800s and found that bees in small cell-size brood combs were smaller than that in standard brood combs, but the decrease in a bee’s body size was not in proportion to the decrease in cell size [8]. Zhang et al. used combs from *Apis mellifera* to rear *Apis cerana*, and the weight of adult worker bees was significantly more than the bees from natural combs [9]. Recent research revealed that the inner cell diameter gets smaller with increasing comb age, and the worker bees that emerge from older combs have lighter body weights and smaller body sizes [10]. The development of worker bees and the queen bee are both influenced by cell size. Queen bees reared in large queen-cell cups have wider thorax and abdomen widths, which positively affects their weight [11]. In addition, it was reported in an earlier study that small cell size can impact *Varroa* mite reproduction, potentially reducing success, leading to higher nonreproduction rates, delayed reproduction, and male absence [12]. Another study reported that, however, this effect may not be consistent across all populations. In surviving colonies, smaller cell size does not seem to impact mite reproductive success, suggesting that other mite-surviving mechanisms are favored by natural selection [13]. The small size of the cells helps trigger the hygienic behaviors of the colony [14]. Overall, the relationship between cell size and mite reproduction is complex and may depend on various factors such as the presence of specific traits in the worker bee population [15]. It has also been reported that the large size of the cells can reduce the reproduction rate and fecundity of *Varroa destructor* [16].

Recently, 3D-printing technology has been used in various industries. To figure out the influences of cell size on the development process of drone bees, we used 3D-printing technology to design and construct experimental combs with different sizes of foundations according to our experimental design, and we measured the body size and weight of drone bees at different developmental stages (including head width, chest width, abdominal width, body weight, and body length). The study aimed to understand the effect of different cell sizes on the drone’s morphological characteristics and to verify if the bee colony can accept the 3D-printed foundations. Although the materials used differ from beeswax, this method allows for the creation of desired cell sizes. The study also aims to determine if worker bees can construct complete cells from the foundation.

## 2. Materials and Methods

### 2.1. Honey Bees

This experiment was carried out between April and June 2022. The drones were reared in the honeybee (*Apis mellifera ligustica*) colony at the experimental apiary of the Institute of Zoology, Guangdong Academy of Sciences, Guangzhou, China (23°5′44″ N, 113°17′17″ E). The colony was housed in standard Langstroth hives and was healthy, without infestation by *Varroa destructor.*

### 2.2. Design and Use of Artificial Comb Foundations

Comb foundation models with different cell widths, including 5.0 mm, 5.5 mm, 6.0 mm, 6.5 mm, and 7.0 mm, were designed using SolidWorks 2016 software (Dassault Systèmes, Waltham, MA, USA). In *Apis mellifera* colonies, the size of the worker cell is 5.20–5.40 mm, while the width of the drone cell is 6.20–6.40 mm. So, we chose a cell width of 5.00 to 7.00 mm with 0.5 mm intervals, which includes the cell dimensions of both the worker and drone [17]. The cell width refers to the internal distance between opposite sides of a hexagonal cell. The cell-wall thickness and cell depth were 0.5 mm and 1.48 mm. The length, width, and thickness of each foundation were 133.0 mm, 91.0 mm, and 3.2 mm, respectively. All artificial comb foundations were manufactured by using 3D-printing technology, and Wenext R4600 photosensitive resin (Wenext, Shenzhen, China) was used as the printing material. Then, a thin layer of pure beeswax was coated on the artificial foundations. Five artificial foundations of different cell sizes and one commercial standard worker foundation (cell width: 5.0 mm) were assembled to form a regular frame and then inserted into a strong honeybee colony to let the bees draw out the foundations.

### 2.3. Measurement of Cell Size

The cell width of each comb built up on the foundations of different cell sizes was measured to evaluate the deviation between the expected and actual cell sizes. Select 40 random nests from the comb of each cell size and measure the straight-line distance between the medial sides of any two opposite sides of the hexagonal nest using a digital vernier caliper (Deli, Deli tools, Ningbo, China). The foundation dimensions are measured using digital vernier calipers and are exactly the same as the design dimensions of the foundation model. To reduce the error, the measurement of cell-wall thickness requires the outer measuring stick of the vernier caliper to be completely attached to the cell wall. The cell length, cell width, and cell thickness of the foundation are also the same; the difference is that the inner measuring stick of the vernier caliper is used to measure the cell depth and cell width. Subsequently, the internal distance between the opposite sides of the hexagonal cell is measured, and the inner wall of the cell refers to the depth of the connection from the inner wall of the cell to the bottom of the cell.

### 2.4. Morphological Analysis of Drone Brood

The frame with combs of different cell sizes was returned to the same colony to allow the queen bee to lay eggs. Only worker eggs were laid in 5.0 mm wide cells, and both worker and drone eggs were produced in 5.5 mm wide cells, while only drone eggs were found in cells of larger sizes. We collected drone brood from 6.0 mm, 6.5 mm, and 7.0 mm wide cells and measured several morphological indicators, such as body weight, body length, head width, thorax width, and abdomen width. Different developmental stages of drone brood were sampled, including LS (5th instar larva after sealing), Pw (pupa with white eyes), Pd (pupa with dark-brown eyes), Pdl (pupa with dark-brown eyes and lightly pigmented thorax), Pdm (pupa with dark-brown eyes and medium colored thorax), and newly emergent adults. The developmental stages were characterized according to the previous studies [18,19]. At least 20 drones were collected randomly from each comb at each stage. Then, the weights of the drones were measured with an electronic balance (Sartorius, Sartorius Group, Göttingen, Germany). Furthermore, other morphological indicators of drones, including head width, thorax width, abdomen width, and body length, were measured with a digital vernier caliper.

### 2.5. Statistical Analysis

A one-sample *t* test was used to test the differences between the actual cell sizes and the theoretical values. For the data on the morphological indicators of drones, the Brown–Forsythe test was used to analyze the variances of all groups to ensure homogeneity of the variances. Then, an ANOVA was performed on the data to determine the significant differences, followed by Tukey’s HSD test for post hoc comparisons. A linear regression was conducted to parameterize the relationship between cell size and body weight in the drones. All the analyses were conducted using GraphPad Prism software version 7.0 (GRAPH PAD Software Inc, San Diego, CA, USA).

## 3. Results

### 3.1. The Drawing out of the 3D-Printed Foundations and Brood Rearing

**Figure 1:**
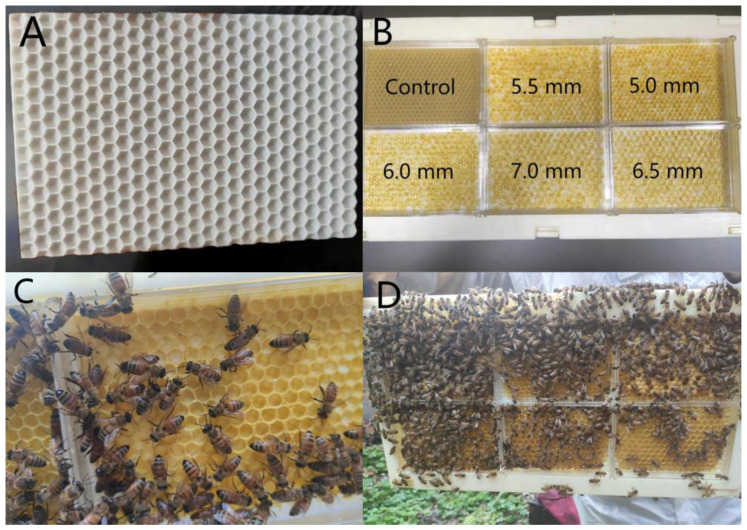
Design and use of artificial comb foundations. (**A**) A 3D-printed comb foundation. (**B**) Coating and assembling of the foundations of different sizes. The cell width is labeled on each foundation. One commercial worker foundation is included as control. (**C**) Drawing out of wax on the foundations. (**D**) Brood raising in the building-up cells.

As shown in Figure 1, honey bees were able to draw out the wax and build up full cells on the 3D-printed foundations, just as they were able to on the control wax foundation, and the actual cell sizes were generally kept the same as desired, even though a slight decrease was found in the cell size on the 6.5 mm and 7.0 mm foundations (Table 1, one-sample *t* test: for 6.5 mm, *p* < 0.001; for 7.0 mm, *p* < 0.0001).

Regardless of the cell sizes, the queen bee was able to lay eggs inside, and, subsequently, the worker bees would raise the hatched eggs, as depicted in Figure 1D. Interestingly, the sex of the offspring was adjusted according to the different cell sizes. Only female bees (workers) were produced in the combs of smaller cell size (5.0 mm), while males (drones) were generated in the combs of larger cell size (6.0 mm, 6.5 mm, and 7.0 mm). As for the medium-sized cells (5.5 mm), both the worker and drone broods were randomly scattered over the comb.

### 3.2. Morphological Measurement of Drone Development

**Figure 2:**
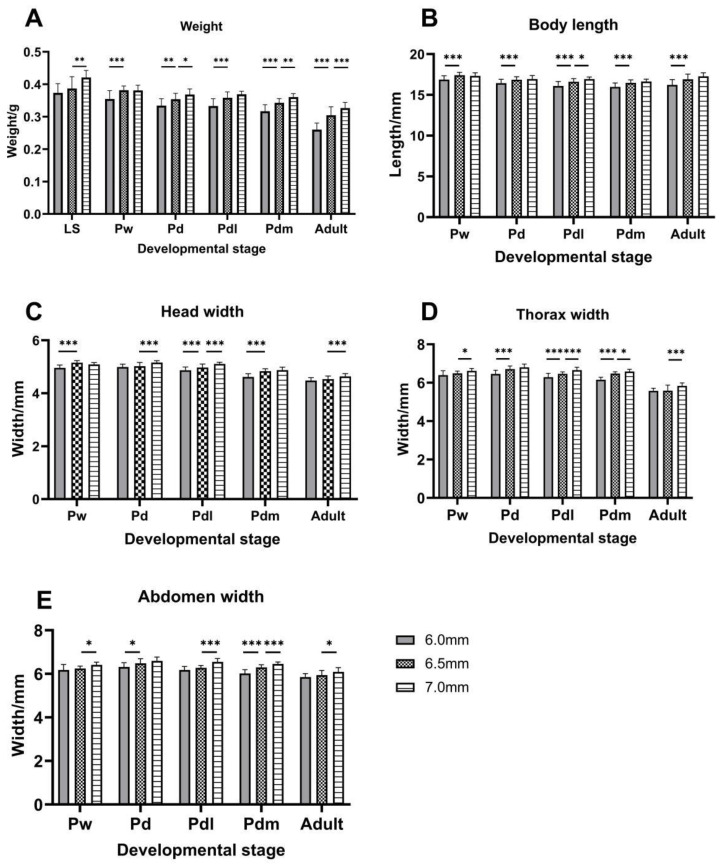
Morphological measurement of drones at different developmental stages in three cell size combs. LS, 5th instar larva after sealing; Pw, pupa with white eyes; Pd, pupa with dark-brown eyes; Pdl, pupa with dark-brown eyes and lightly pigmented thorax; Pdm, pupa with dark-brown eyes and medium colored thorax; Adult, newly emergent drones. Values are presented as mean ± SD. The sample size range from 20 to 44. One-way ANOVA followed by Tukey’s multiple comparison test is used to determine the significant differences between all data sets. * *p* < 0.05, ** *p* < 0.01, *** *p* < 0.001.

At the LS stage, the body weight of the drone in the 6.5 mm cell was higher than in the 6.0 mm cell but was not significant, and, in the 7.0 mm cell, the weight was significantly higher than it in the 6.5 mm cell (Figure 2A. ANOVA, F (2, 57) = 13.82, *p* < 0.0001, Tukey’s HSD test, *p* = 0.0016). The drones were heavier in the larger-sized cells during the pupae stage and adult stage, and the body weight of the drone in the 6.5 mm cell was significantly higher than that in the 6.0 mm cell (Tukey’s HSD test: Pw, *p* < 0.0001; Pd, *p* = 0.0037; Pdl, *p* < 0.0001; Pdm, *p* < 0.0001; Adult, *p* < 0.0001). It was significantly higher in the 7.0 mm cell than 6.5 mm cell at the Pd, Pdm, and adult stages (Tukey’s HSD test: Pd, *p* = 0.0463; Pdm, *p* = 0.0010; adult, *p* = 0.0004).

In addition, the drone’s head width was significantly wider in the 6.5 mm cell than that in the 6.0 mm cell at the Pw stage, Pdl stage, and Pdm stage (Tukey’s HSD test: Pw, *p* < 0.0001; Pdl, *p* = 0.0076; Pdm, *p* < 0.0001), and it was significantly wider in the 7.0 mm cell than that in the 6.5 mm cell at Pd stage, Pdl stage, and adult stage (Tukey’s HSD test: Pd, *p* = 0.0004; Pdl, *p* = 0.0004; adult, *p* = 0.0027) (Figure 2C). 

It is obvious that the thorax width of the drone was wider when they had thrived in the larger-sized cells among all stages in Figure 2D, and it was significantly wider in the 7.0 mm cells than the 6.5 mm cells at all stages, except the Pd stage (Tukey’s HSD test: Pw, *p* = 0.0419; Pdl, *p* = 0.0001; Pdm, *p* = 0.0109; Adult, *p* = 0.0001). At the Pd, Pdl, and Pdm stages, the thorax width of a drone was also significantly higher in the 6.5 mm cell than 6.0 mm cell (Tukey’s HSD test: Pd, *p* < 0.0001; Pdl, *p* = 0.0008; Pdm, *p* < 0.0001). 

Meanwhile, drones in larger-size cells had a widened abdomen in Figure 2E. The results show that the abdomen width of the drone in the 6.5 mm cell was significantly wider than that in the 6.0 mm at the Pd and Pdm stages (Tukey’s HSD test: Pd, *p* = 0.0247; Pdm, *p* < 0.0001), and at the Pw stage, Pdl stage, Pdm stage, and adult stage, the drone’s abdomen width in the 7.0 mm cell was significantly wider than that in the 6.5 mm cell (Tukey’s HSD test: Pw, *p* = 0.0123; Pdl, *p* < 0.0001; Pdm, *p* = 0.0008; adult, *p* = 0.0105). 

The body length of the drone was also longer when they were breeding in larger-size cells (Figure 2B). The body length of the drone in the 6.5 mm cell was significantly longer than that in the 6.0 mm cell at every stage (Tukey’s HSD test: Pw, *p* = 0.0003; Pd, *p* = 0.0066; Pdl, *p* = 0.0003; Pdm, *p* = 0.0002; adult, *p* < 0.0001), but comparing the 6.5 mm cell to the 7.0 mm cell, the body-length data were not significant except in the Pdl stage (ANOVA, F (2, 63) = 22.89, *p* < 0.0001, Tukey’s HSD test, *p* = 0.0207). 

### 3.3. The Relationship between Cell Size and the Body Weight of Drones

**Figure 3:**
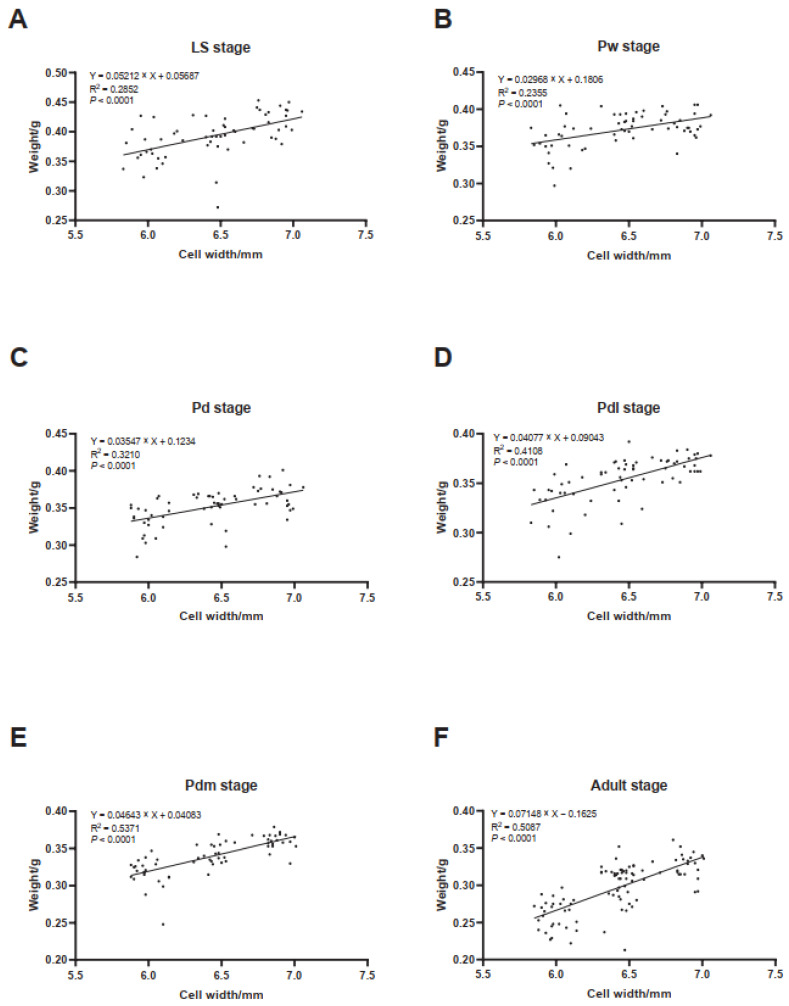
Linear regression of body weight of drones on comb cell size. The equation, R squared value, and *p* value are displayed. LS, 5th instar larva after sealing (**A**); Pw, pupa with white eyes (**B**); Pd, pupa with dark-brown eyes (**C**); Pdl, pupa with dark-brown eyes and lightly pigmented thorax (**D**); Pdm, pupa with dark-brown eyes and medium colored thorax (**E**); adult, newly emergent drones (**F**).

As shown in Figure 3, no matter the developmental stage, the body weight of drones linearly increased with the sizes of comb cells (simple linear regression; Figure 3A, the LS stage, slope = 0.052, *p* < 0.0001; Figure 3B, the Pw stage, slope = 0.030, *p* < 0.0001; Figure 3C, the Pd stage, slope = 0.035, *p* < 0.0001; Figure 3D, the Pdl stage, slope = 0.041, *p* < 0.0001; Figure 3E, the Pdm stage, slope = 0.046, *p* < 0.0001; Figure 3F, the adult stage, slope = 0.071). 

### 3.4. The Morphological Changes of Drones in Differently Sized Comb Cells

**Figure 4:**
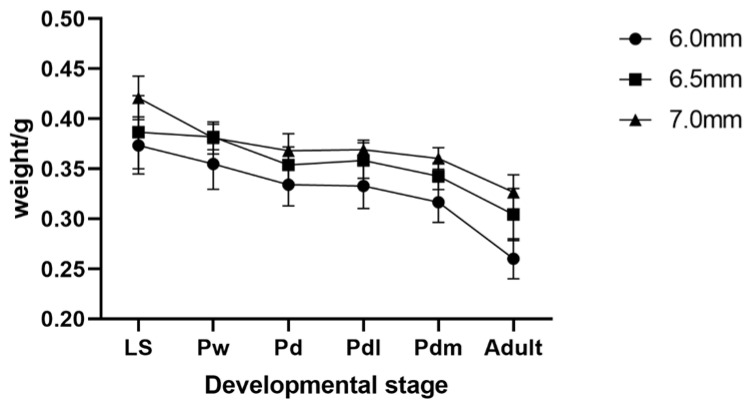
The change of body weight of drones from different cell-sized combs. Values are presented as mean ± SD. LS, 5th instar larva after sealing; Pw, pupa with white eyes; Pd, pupa with dark-brown eyes; Pdl, pupa with dark-brown eyes and lightly pigmented thorax; Pdm, pupa with dark-brown eyes and medium colored thorax; Adult, newly emergent drones.

During the developmental stages, only the weight data have obvious changes from the LS stage to the adult stage, as shown in (Figure 4). The average body weight of drones in the 6.0 mm cell-size combs was 0.373 ± 0.03 g, decreased to 0.260 ± 0.02 g from the LS stage to the adult stage, reduced 30.31% from 0.387 ± 0.04 decrease to 0.304 ± 0.03 in 6.5 mm cell-size combs, reduced 21.30%, and from 0.421 ± 0.02 decrease to 0.327 ± 0.02 in 7.0 mm cell-size combs, reduced 22.41%. Compare the weight of drones of any one of the developmental stages between the 6.0 mm and 6.5 mm cell-size combs or the 6.5 mm and 7.0 mm cell-size combs. The weights of drones in large cell-size combs were about 3~8% heavier than that in the small cell-size combs, except for two stages, where the rate was 0.10% at the Pw stage between 6.5 mm and 7.0 mm. In adult stages, the weights of drones in the 6.5 mm cell-size combs were about 16.99% heavier than that in 6.0 mm cell-size combs. In contrast, the other morphological changes of drones in different developmental stages were out of proportion to the changes in cell size, resulting in smaller-size cells having a higher fill factor (thorax width/cell width), as shown in Table 2.

## 4. Discussion

This study aims to analyze the effect of comb cell size on the morphology of drones by using an artificial comb. In previous studies, people used commercial foundations or foundations of other species of bees as standard foundations and large foundations [7,8,20]. In this experiment, we used 3D-printing foundations made of white resin with the size we designed. Our result shows that the final artificial comb meets our expectations, and the bees could reproduce normally. The combs that the bees built were almost conformed to the size of the foundations, except for the sizes of 6.5 mm and 7.0 mm.

The start of comb construction by honeybees is affected by many factors, such as temperature and climate; bees need to build new combs to solve their dilemma according to the condition of the colony and environment. While there is adequate nectar and pollen in the field, and the comb is full of nectar, bees will start to build new combs [21]. Our experiment was carried out during the breeding season with enough nectar and pollen in the field; the honey was full in the comb and the attainment reached its fullness threshold in the colony. Thus, our artificial comb was constructed by honeybees successfully. Bees also regulate the relative number of worker bees and drone bees by building combs with different cell sizes, which is vital to the investment in female reproduction [22]. In the natural honeybee colony, the smaller size of the comb (approximately 5.2 mm) is used to rear workers, and the larger size of the comb (approximately 6.2 mm) is used to rear drones [23]; there is an upper limit to the size of the comb for breeding drones [24]. In our experimental combs, the small cell-size combs were in keeping with the foundations, but there was a deviation between the larger cell-size combs and the foundations. In our experiment, the 6.5 mm and 7.0 mm foundations may be too large for bees to construct, and the final cell size was a deviation from the foundations. Bees start to build drone cells during breeding season and can regulate their construction well to build the correct type of combs they need. Workers will directly contact the drone comb to generate negative feedback to inhibit the construction of further drone combs [23]; therefore, the area of the drone comb is usually less than one-quarter of the total comb area. In our experiment colony, there were already too many drone cells, and the colony needed more workers rather than drones, which reduced the desire of bees to build drone combs. The bees even added wax to the cell walls to reduce the inner diameter of cells to be approximated to worker cells [25]. In addition, the comb construction is not completed by a single bee; comb construction is a highly distributed activity, and workers start building the comb at multiple locations concurrently; all the bees must agree on what kind of cell to build to keep the comb coherent [24,26]. Thus, the combs on the large-cell-size foundations constructed by the bees in our experimental colony, which had the full desire to build worker combs, have deviated from the ideal size. These also affect bees when building drone combs on the 6.5 mm and 7.0 mm foundations.

In this research, there are significant differences in the morphology of drones from the 6.0 mm, 6.5 mm, and 7.0 mm combs. The drones from the 7.0 mm cell-size combs were obviously larger than those from the smaller cell-size combs, and the sizes of drones in the 6.5 mm cell-size combs are also larger than those in the 6.0 mm cell-size combs. These results are in agreement with McMullan et al. [7] and Grout [8]; these previous studies documented that the morphology of worker bees is affected by the size of combs and small-cell brood combs resulted in smaller bees. Our results confirm that the morphology of drones is affected by the size of the combs as well as the workers. Grout concluded that the increases in the body size of worker bees are proportional to the percentage increases in the brood cell size [7], but McMullan et al. said that the size reduction was clearly not in proportion to the decrease in cell size. In his study, the cell size of the comb was reduced by 7–8%, resulting in only a 1% reduction in the linear body size of bees [8]. Our results are similar to McMullan’s; in our research, we compare the 6.0 mm cell-size comb to the 6.5 mm cell-size comb and the 6.5 mm cell-size comb to the 7.0 mm cell-size comb. The average data in the large cell-size comb was 7.52% and 6.54% bigger than that in the smaller cell-size comb, but the morphological change rates of drones were only 1~4% among all stages between three different cell sizes of combs. Only the change rate of weight (3~8%) was close to that of the cell size of the combs. The average weight of drones decreased by 30.31% from the LS stage to the adult stage in 6.0 mm cell-size combs, and the rate was bigger than that in other cell-size combs. The small cell-size comb may limit the development of drones; thus, there is space limitation and the relative reduction of food [10].

During the pupa stage, the change in comb cell size had little effect on the body size of drones. With the passing of time, the morphological changes of drones in three different cell-size combs were consistent. The body-size reduction of drones caused by cell-size reduction is a step change rather than a response that changes with time. In our research, the change in body size of drones caused by the change in cell size took place as early as the larval stage; this may be related to the amount of food eaten by larvae. The body size of the bumblebee shows relatively high variation within the colony on account of the larval nutrition. Nurses feed the larvae regularly to provide nutrition for them to grow up, and the worker larvae enter their final larval stage with roughly the same weight. However, their weight will vary according to the amount of food they eat at the prepupal stage, which is largely due to the difference in the spatial distribution of larvae and nurse bees [27]. At present, the influence of comb cell size on larval food intake has not been clarified, and further experiments are needed.

Body size affects worker bees’ flying ability and foraging distance; larger bees have larger foraging distances than smaller bees [28]. The bumblebees have a similar situation; large workers possess larger eyes, large brains, and stronger circadian rhythms, which make them more suitable for foraging [29]. Our experimental comb-emerged drones with different body sizes. The primary role of drones in the colony is mating, and relatively large males have advantages in the male competition of some species [30]. The body size of male individuals will directly affect the mating success rate of bumblebees [31], and body size also affects the mating success of honeybees. The flying activity of larger drones reached its peak at noon, which is consistent with the peak of the queen’s activity, while the small drones flew at times of the day when fewer queens were available. The lower mating success of small drones may be due to their weak flight ability [32]. In addition to mating behavior, sperm quality is also a key factor that influences reproduction in the colony; previous studies have shown that large drones produce more spermatozoa than small ones and have more advantages in mating [33,34]. Using combs with different cell sizes can produce drones with different body sizes, which is conducive to researching drones. In the future, further experiments could be conducted to explore the effects of different drone sizes on queen fecundity.

The ectoparasitic mite *Varroa destructor,* which caused damage to *Apis mellifera* worldwide, reproduces in the worker cells and drone cells, and the comb’s cell size affects the infestation rate and reproduction of mites. The relationship between cell size and the reproduction of mites has been studied a lot, but earlier studies were conflicting. One study suggested that small-size cells have a role in the resistance of bees to the infestation of *V. destructor* [35]; Maggi et al. found that the infection rate of *V. destructor* increased linearly with the width of cells of workers and drones and found more infertile mites in the small-size cells [36]. Other researchers show that cell size has no significant effect on the reproductive success rate of *V. destructor*, and 4.8 mm size cells attract more mites than larger cell-size combs [37], and small cell-size combs do not inhibit mite reproduction [15,38]. A recent study did not examine the effect of cell size on the reproductive success of mites, but the data showed that mites had significantly lower reproductive success in small-sized cells [13]. We consider that the influence of comb cell size on mite reproduction mainly rests with the change of cell space and fill factor. Martin found that the mites produced fewer fertilized females in the cells with pseudo-clone bees than in normal cells because the pseudo-clone was 8% larger than their host, and, therefore, the fill factor was reduced [39]. Seeley and Griffin suggested that small-sized cells do not inhibit mite reproduction on account of the fill factor, which was almost the same in small cells and standard cells [15]. Our research did not measure the inner diameter of cells, so we did not figure out the fill factor of three cell-size combs. However, the thorax and abdomen data of drones show significant differences between the three groups. We found the change in the body size of drones was far less than the change in comb cell size, and they were out of proportion, which means smaller cell-size cells or larger cell-size cells have more or less fill factor. This may be the reason that some studies believe that smaller cell-size combs are beneficial in inhibiting the reproduction of *V. destructor*. It was noted that Zhou et al. found *V. destructor* reproduced better in worker cells than drone cells when there were worker larvae reared in drone cells. She and her team inferred that larger or smaller cell-size combs will inhibit the reproduction of *V. destructor* from different aspects [16]. Studies on the relationship between comb cell size and the reproduction of mites need more experiments to prove. 

In addition, the availability of *Varroa* mites for experiments depends on season and climatic conditions, and there is no method to cultivate mites in vitro in the laboratory at present [40]. The mites reproduce mainly in the drone cells, and adding large cell-size combs to the colony in breeding season will help to breed more drones in a short time, which is conducive to breeding more mites for experimental research. However, it is adverse to the development of the colony.

## 5. Conclusions

Our study suggests that morphological characteristics, such as weight, body length, head width, thorax width, and abdominal width, increase with cell size, similar to those reported in worker and queen bees. These results reflect the importance of cell size for drone development; that is, by artificially changing the cell size, drones of different sizes can be bred. In particular, the weights of drones, regardless of stage, are linearly positively correlated with cell size. Other body morphological traits also disproportionately increase as the cell size increases. In particular, our findings provide a new way to fabricate cells of different sizes, and the application of 3D-printing technology can help us build size-accurate foundations. In conclusion, our findings and previous studies suggest that the morphological size of worker bees and drones is dependent on cell size. However, cell size is only an important reason, and a better understanding of the correlation between cell size and morphological changes in honeybees is essential to understanding the underlying regulators of morphological changes in honeybees.

## Figures and Tables

**Table 1:** Measurement of cell sizes of combs drawn out on the artificial foundations.

Group	Cell Width of Foundation/mm	Cell Width of Drawn-Out Comb/mm	Significance
Control	5.00	5.00 ± 0.02	a
5.0 mm	5.00	5.01 ± 0.03	a
5.5 mm	5.50	5.50 ± 0.02	a
6.0 mm	6.00	6.00 ± 0.10	a
6.5 mm	6.50	6.45 ± 0.08	b
7.0 mm	7.00	6.87 ± 0.09	b

The data are presented as mean ± SD; a: means not significant; b: *p* < 0.001.

**Table 2:** The fill factor of three groups at the adult stage.

Group	Thorax Width/mm	Cell Width of Drawn Out Comb/mm	Fill Factor/%
6.0 mm	5.58 ± 0.13	6.00 ± 0.10	92.9
6.5 mm	5.58 ± 0.28	6.45 ± 0.08	86.6
7.0 mm	5.84 ± 0.15	6.87 ± 0.09	85.0

## Data Availability

Data are contained within the article.
